# Salmon lice in the Pacific Ocean show evidence of evolved resistance to parasiticide treatment

**DOI:** 10.1038/s41598-022-07464-1

**Published:** 2022-03-28

**Authors:** Sean C. Godwin, Andrew W. Bateman, Anna Kuparinen, Rick Johnson, John Powell, Kelly Speck, Jeffrey A. Hutchings

**Affiliations:** 1 Department of Biology, Dalhousie University, Halifax, NS Canada; 2 Salmon Coast Field Station, Simoom Sound, BC Canada; 3 Pacific Salmon Foundation, Vancouver, BC Canada; 4 Department of Biological and Environmental Science, University of Jyväskylä, Jyvaskyla, Finland; 5 Kwikwasut’inuxw Haxwa’mis First Nation, Gilford Island, BC Canada; 6 Mamalilikulla First Nation, Campbell River, BC Canada; 7 ‘Na̱mg̱is First Nation, Alert Bay, BC Canada; 8 Institute of Marine Research, Flødevigen Marine Research Station, His, Norway; 9 Centre for Coastal Research, University of Agder, Kristiansand, Norway

**Keywords:** Conservation biology, Marine biology

## Abstract

Parasitic salmon lice (*Lepeophtheirus salmonis*) threaten the economic and ecological sustainability of salmon farming, and their evolved resistance to treatment with emamectin benzoate (EMB) has been a major problem for salmon farming in the Atlantic Ocean. In contrast, the Pacific Ocean, where wild salmon are far more abundant, has not seen widespread evolution of EMB-resistant lice. Here, we use EMB bioassays and counts of lice on farms from the Broughton Archipelago, Canada—a core region of salmon farming in the Pacific—to show that EMB sensitivity has dramatically decreased since 2010, concurrent with marked decrease in the field efficacy of EMB treatments. Notably, these bioassay data were not made available through public reporting by industry or by the federal regulator, but rather through Indigenous-led agreements that created a legal obligation for salmon-farming companies to provide data to First Nations. Our results suggest that salmon lice in the Pacific Ocean have recently evolved substantial resistance to EMB, and that salmon-louse outbreaks on Pacific farms will therefore be more difficult to control in the coming years.

## Introduction

Salmon farming is one of the most valuable forms of aquaculture worldwide^1^, but its economic and ecological sustainability is continuously threatened by the salmon louse (*Lepeophtheirus salmonis*)^2^. This ectoparasitic copepod transfers between farmed and wild salmon throughout the northern hemisphere, and can impact hosts at the cellular, individual, and population levels^2^. In many parts of the world, the preferred method of control for salmon lice has been an in-feed parasiticide called emamectin benzoate (EMB; trade name SLICE^®^)^3^, but rapidly evolved resistance has contributed to the chemical being used more sparingly and in combination with many other treatments in Atlantic-Ocean farming regions^4–6^.

In stark contrast to the situation in the Atlantic, salmon lice in the Pacific Ocean appeared to have avoided widespread evolution of EMB resistance^7^ despite EMB being virtually the only treatment option used in the region until roughly 2017. Canada is the main salmon-farming country in the north Pacific, and genetic evidence has revealed only localized ephemeral resistance here^8^, potentially due to the large wild Pacific salmon populations that act as untreated refuges for susceptible lice^7, 9^. Pacific Canada is unique globally for having substantial populations of *both* farmed and wild salmon^1^. Management decisions for salmon aquaculture in Pacific Canada are thus unmatched worldwide for their potential impacts on the natural systems that depend on salmon.

Resistance to EMB is typically identified through bioassays in which hundreds of salmon lice, grouped by sex, are placed in baths of different concentrations along a gradient of EMB for 24 hours^10^, which differs from the host-dependent exposure that lice on farms experience due to in-feed administration of EMB. For each bioassay, the effective concentrations at which 50% of lice survive (EC_50_) are calculated, and if EC_50_ values increase over time then EMB resistance in salmon lice is inferred. Male salmon lice are generally more resistant to EMB than females, for reasons that are unclear^11^. Salmon-farming companies in Pacific Canada regularly conduct bioassays to assess EMB sensitivity of lice on their farms, but the raw data have never been publicly available. Summarized bioassay data were last made available in 2012^10^.

**Figure 1:**
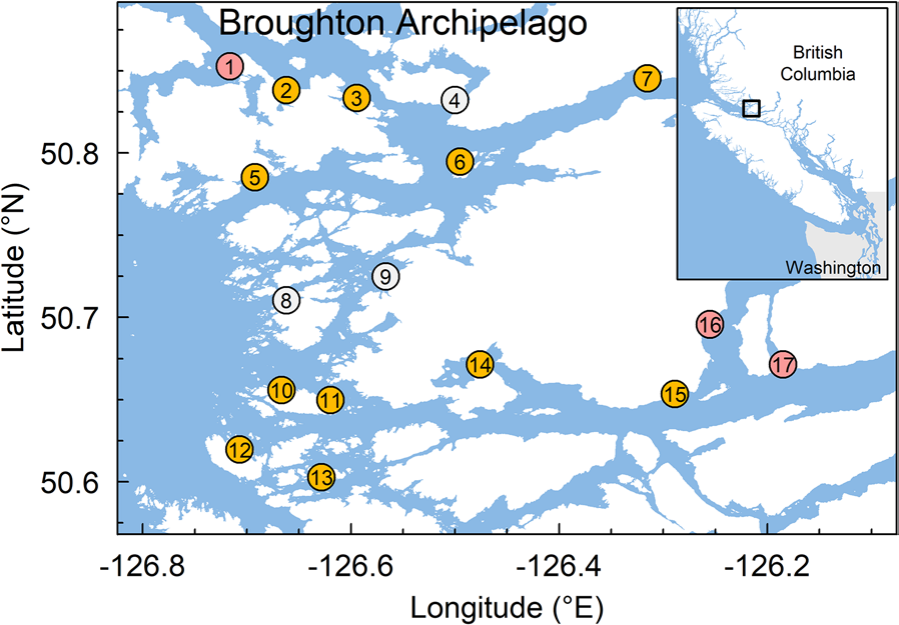
Map of salmon farms active between 2010 and 2021 in the traditional territories of the Mamalilikulla, ‘Na̱mg̱is, and Kwikwasut’inuxw Haxwa’mis First Nations in the region now known as the Broughton Archipelago, British Columbia, Canada. Orange points represent farms that performed at least one bioassay, pink points are farms that performed EMB treatments but no bioassays, and grey points are farms that performed neither EMB treatments nor bioassays (between 2010 and 2021). Numbers correspond to the Farm IDs in Table S1. The spatial extent of the main panel is indicated by the black rectangle in the inset.

Here, we assess whether Pacific salmon lice have evolved EMB resistance by analyzing bioassay, treatment, and salmon-louse count data from 2010 to 2021 in the Broughton Archipelago (BA), British Columbia (BC) (Fig. 1), long a focus of salmon-farm research and management in the Pacific^12^.

## Results

**Figure 2:**
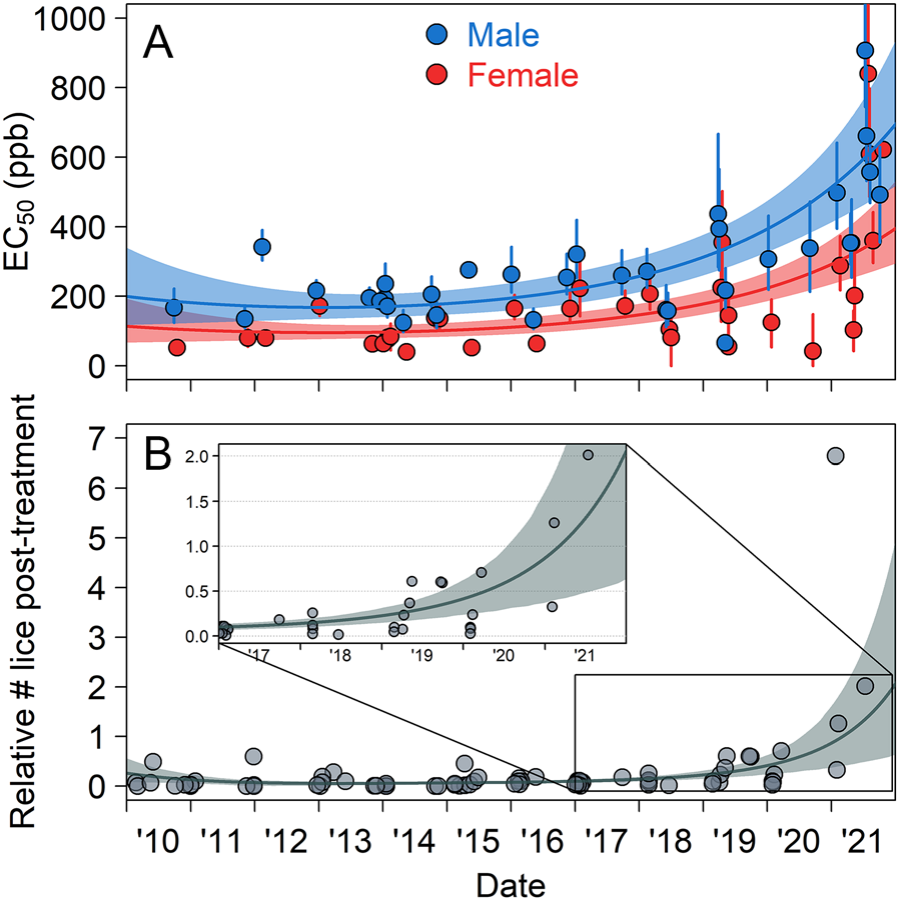
Increasing trends in: (**A**) salmon-louse resistance to emamectin benzoate (EMB), and (**B**) relative salmon-louse counts after EMB treatment. Points in panel (**A**) represent the effective concentrations required to kill 50% of male (blue) or female (red) salmon lice (EC_50_) in bioassays. Points in panel (**B**) show the relative post-treatment counts (i.e., the post-treatment counts divided by the pre-treatment counts). Lines depict the mean predictions from the top-ranked models. Error bars and shaded regions give 95% confidence intervals.

Salmon lice collected from BA salmon farms exhibited a decline in EMB sensitivity over time (2010 to 2021). The highest EC_50_ observed was 907 (95% CI: 744, 1189) ppb for male lice and 840 (695, 1073) ppb for females (Fig. 2A) from a bioassay performed in July 2021. These EC_50_ values constitute a fivefold increase for males and a 16-fold increase for females compared to the EC_50_ values from the initial bioassay at the same farm in 2010 (approximately 40–50 louse generations). Our most parsimonious description of the EC_50_ data, which included effects for sex and previous treatment and a quadratic effect for time, indicated a drastic increase in EC_50_ over the past few years (Fig. 2A). Previous treatment increased EC_50_ values by a factor of 1.23 (0.95, 1.59). We found no support (0% by Akaike weight) for EMB sensitivity remaining constant over time.

Field efficacy of EMB also decreased over time, with salmon-louse counts on farms displaying reduced responses to treatment in recent years. Prior to 2019, post-treatment counts were 8.6% ± 1.6% (mean ± SE) of pre-treatment counts (Fig. 2B), and only one of the 56 relative post-treatment counts (1.8%) was over 0.5. In contrast, between 2019 and 2021, seven of the 17 relative counts (41%) were over 0.5. The three least effective treatments all occurred in 2021, and all three resulted in higher counts post-treatment than pre-treatment, suggesting treatment failure. The most parsimonious description of the relative-count data (i.e., the post-treatment counts divided by the pre-treatment counts) was one that included an effect for previous treatment and a quadratic effect of time. Relative post-treatment counts increased dramatically in recent years (Fig. 2B), signifying a severe, recent decrease in treatment efficacy. Previous treatment increased non-zero relative post-treatment counts by a factor of 2.18 (1.12, 4.24). We found no support (0% by Akaike weight) for relative post-treatment counts remaining constant over time.

## Discussion

Salmon lice in the Pacific Ocean appear to have evolved EMB resistance based on two lines of evidence. First, lice from BA salmon farms experienced decreased sensitivity to EMB in bioassays conducted between 2010 and 2021. Second, field efficacy of EMB treatments on these farms declined over the same time period. The five highest EC_50_ values for males (492–907 ppb) and the four highest for females (360–840 ppb) all occurred in 2021; these are comparable to, or higher than, those for treatment-resistant strains of salmon lice in Atlantic Canada (329–840 ppb for males and 170–304 ppb for females)^3, 6, 11^. Importantly, these data, and therefore our findings, were not shared via public reporting by industry or the federal regulator, but instead through an Indigenous-led monitoring agreement.

The apparently resistant salmon lice from the recent bioassays are obvious candidates for follow-up genetic analyses. Such genetic work is beyond the scope of the present study, in part because the lice were not retained by the salmon-farming companies to the First Nations. Although there are still no diagnostic genetic tests for EMB resistance of Pacific salmon lice, a rare louse genotype (with characteristic single nucleotide polymorphisms) was recently linked to ephemeral EMB tolerance in BC^8^. These signs of nascent resistance, evident in samples from nearly a decade ago, strongly suggest that the trends we document here have a genetic basis and are not merely plastic changes. Our results highlight the need for assessments of the frequency of this rare genotype, ideally with full public reporting and independent verification, as an integral part of EMB bioassays until a full diagnostic test is developed.

While this is the first published account with evidence for the evolution of EMB resistance in the Pacific Ocean, it seems quite likely that industry and the federal regulator have been aware of this emerging issue for some time. EMB tolerance was reported in 2013 as a localized and short-lived phenomenon in one farm in BC^10^, and again in 2018^8^, which presumably raised concerns internally about resistance becoming a pervasive problem. Treatment failures^8, 13^, alternative treatments^8, 13^, persistently elevated counts^14^, and concerning bioassays^8^ have occurred in other parts of Pacific Canada at earlier dates than in the BA. Combined with our findings, this suggests that EMB resistance is widespread and well established in BC. Whether large returns of wild salmon could impart some relief to farms by providing an influx of treatment-susceptible lice will be a situation to monitor over the coming years, but is probably unlikely given the trends we report here across 11 farms.

Despite the local and global forewarnings of EMB resistance, industry was exclusively permitted to use EMB for delousing treatments in the BA until late 2019, a strategy which imposes strong selection and likely resulted in accelerated evolution of resistance^15^ (but see^7, 9^). Three additional treatment options (i.e., freshwater baths, hydrogen peroxide baths, and jets of pressurized water) have since been introduced and are now used frequently; these other treatments accounted for 62% of treatments in 2021. With EMB effectiveness declining, industry has had to (and will continue having to) reactively (rather than proactively) diversify its approaches to louse control in order to better align with the integrated pest management strategies used in other countries. A suite of non-chemical preventative methods (e.g., barriers to limit surface interactions between fish and lice) have had promising results elsewhere, and many other chemical, mechanical, and biological treatment options are also available (see review by Coates et al.^15^). Each of these treatment options has its own drawbacks, however (e.g., resistance to chemotherapeutants^4, 15^, welfare issues from mechanical treatments^16^, and pathogen transmission with cleaner fish^17^), some of which will worsen with climate change, necessitating a diverse set of strategies for louse control.

Independent scientists have long requested bioassay data from industry and the federal regulator to allow the evidence for EMB resistance to be assessed. Bioassay data have not been publicly released since 2012^10^, and even then they were reported in summarized rather than raw form. The data presented here were made available through legal agreements between First Nations and the relevant salmon farming companies (MOWI Canada West and Cermaq). In 2018, the BC provincial government agreed it would not renew the tenures for 17 fish farms in the Broughton Archipelago unless the Mamalilikulla, ‘Na̱mg̱is, and Kwikwasut’inuxw Haxwa’mis First Nations consented to their renewal. As a result of the First Nations not consenting to these renewals, those 17 fish farms are undergoing an orderly transition from the Broughton Archipelago. As of January 1, 2021, nine of the seventeen farms in the region have been decommissioned. As part of the orderly transition, these three First Nations formed agreements in 2019 with the two salmon-farming companies working in their territories to govern the monitoring, management, and potential removal of the remaining salmon farms over the next few years. These landmark agreements, implemented in accordance with the United Nations Declaration on the Rights of Indigenous Peoples^18^, mandated the sharing of historical data collected by the salmon-farming companies with these three First Nations. In a province with a long history of industrial exploitation of resources in Indigenous territories^19–21^, these agreements and their outcomes represent a compelling example of Indigenous self-governance that may become more prevalent as First Nations endeavour to gain more control over industrial operations in their traditional territories.

Until recently, the Pacific Ocean was considered the last stronghold of treatment-susceptible salmon lice^7^, but our results suggest that resistance has now emerged. EMB resistance in BC could have arisen from depleted wild salmon populations^7, 22^, ill-advised reliance on a single treatment^15^, or insufficiently aggressive treatment on the part of farms^9, 23^, any of which would suggest a failure of management at some level. Whatever the cause, the emergence of resistant salmon lice in the Pacific poses serious challenges for controlling outbreaks to protect wild salmon in the coming years, further exacerbating the negative consequences of lice on salmon predicted in a warming climate^24^.

## Methods

### Bioassays

Salmon-louse bioassays were performed by the BC Centre for Aquatic Health Sciences (CAHS) as described in Saksida et al.^10^. Briefly, motile (i.e., pre-adult and adult) *L. salmonis* were collected from 11 salmon farms in the Broughton Archipelago (BA) between 2010 and 2021 and transported to CAHS in Campbell River, BC. Within 18 h of collection, healthy lice were separated by sex and randomly placed into petri dishes each containing approximately 10 lice (mean ± SD = 9.6 ± 1.1) and subjected to one of six EMB concentrations (either 0, 31.3, 62.5, 125, 250, and 500 ppb or 0, 62.5, 125, 250, 500, and 1000 ppb, depending on suspected variation in EMB sensitivity^11^). Each collection corresponded to one bioassay, and each bioassay contained roughly four replicates for each sex (4.0 ± 1.3 for females and 3.6 ± 0.9 for males). After 24 h of EMB exposure, lice were classified as alive if they could swim and attach to the petri dish, or moribund/dead otherwise. Lice were kept at 10 °C throughout the process. In total, 34 bioassays were conducted from 11 farms between October 2010 and November 2021.

We analysed the proportion of lice that survived exposure to EMB, using standard statistical descriptions that accounted for within-assay dependencies (generalized linear mixed models (GLMMs) with logit link functions, fitted separately to the data from each bioassay). The models included fixed effects for EMB concentration, sex, and the interaction between the two, as well as a random intercept for petri dish. For each analysis, we centered concentration values and scaled them by one standard deviation. We used the GLMM fits to calculate the effective concentrations at which 50% of the lice survived (EC_50_) in each bioassay. The GLMM for one bioassay produced a singular fit because there was not enough variation in the female survival data to warrant the random-effects structure. We retained the EC_50_ values resulting from this singular fit because re-fitting without the random intercept yielded identical EC_50_ values, and removing the entire bioassay from the overall dataset did not qualitatively affect the subsequent analysis.

To assess whether the sensitivity of salmon lice to EMB has decreased over time, we fitted a set of five standard GLMs with gamma error distributions and log link functions to the maximum-likelihood EC_50_ estimates. Each of these five models included binary effects for sex and for whether the farm’s stock had previously been treated, since both affect EMB sensitivity in lice^10^. The first model included only these two effects and served as a null model that assumed lice did not evolve EMB resistance over time. The second model added a fixed effect for time (i.e., the number of days since January 1, 2010), while the third model included an interaction between time and sex. The fourth and fifth models were identical to the second and third, but with a quadratic effect for time, to account for possible first-order nonlinearity. We were unable to add an effect for farm due to small sample sizes. We performed model selection using the Akaike Information Criterion penalized for small sample sizes AICc^25^, treating AICc differences of less than two as being indistinguishable in terms of statistical support and selecting the least complex model when that was the case^26^. The ΔAICc values for the EC_50_ models were 48.1, 6.1, 4.9, 0, 1.75, respectively.

### Field efficacy

We used relative salmon-louse counts after EMB treatment (i.e., the post-treatment count divided by the pre-treatment count) as our measure of EMB field resistance between 2010 and 2021 (higher relative counts imply lower treatment efficacy). We defined “pre-treatment” as one month prior to treatment and “post-treatment” as three months after treatment (roughly when one would expect to find the lowest counts in louse populations previously unexposed to EMB), as in Saksida et al.^10^. We excluded EMB treatments for which an additional, non-EMB treatment was performed within the following three months. In total, there were 73 EMB treatments for which we were able to calculate relative post-treatment counts.

Salmon-louse counts were performed by farm staff as described by Godwin et al.^27^. In short, salmon-louse counts were usually performed at least one per month by capturing 20 stocked fish in each of three net pens using a box seine net, then placing the fish in an anesthetic bath of tricaine methanesulfonate (TMS, or MS-222) and assessing the fish for motile (i.e., pre-adult and adult) *L. salmonis* by eye.

The treatment dataset included the date and type of every treatment that has been performed on a BA farm (i.e., not just the 11 farms with bioassay data). In total, 88 EMB treatments were conducted between 2010 and 2021, of which we were able to calculate relative post-treatment counts for 73 because some months lacked counts or had a non-EMB treatment performed within the following three months. An additional 22 non-EMB treatments (e.g., freshwater and hydrogen baths) were performed, all since the beginning of 2019, but we excluded these data from our analysis.

To determine whether field efficacy of EMB treatments has decreased over time, we used GLM-based “hurdle models”—standard statistical descriptions used to accommodate an over-abundance of zeroes in data being analysed. A hurdle model uses two components—one model for whether a count is nonzero and another for the value of the nonzero count—to predict overall mean count. To this end, we fitted three binomial GLMs paired with three gamma GLMs to the relative-count data, each of the paired models being structurally identical in terms of predictors. All of these submodels included a binary fixed effect for previous treatment, as in the EC_50_ models. The null pair of submodels included no additional terms, the second pair of submodels included a fixed effect for time (i.e., the number of days since January 1, 2010), and the third pair of submodels included a quadratic effect of time (again, to account for possible first-order deviations nonlinearity). We were unable to add an effect for farm due to small sample sizes. We performed model selection of the hurdle models, again using the Akaike Information Criterion penalized for small sample sizes. The ΔAICc values for the three hurdle models were 39.6, 18.3, and 0, respectively. We performed our analyses in R 3.6.0^28^, using the lme4 package^29^.

## Supplementary Information

Supplementary Table S1.

## Data Availability

All data were provided to the Mamalilikulla, ‘Na̱mg̱is, and Kwikwasut’inuxw Haxwa’mis First Nations by the salmon-farming companies MOWI Canada West and Cermaq as part of the Indigenous Monitoring and Inspection Plan (IMIP) Framework Agreements. The data and analysis code for this study are available in an open-access GitHub repository found at 10.5281/zenodo.6341974.
