# Supplementary material for: Salmon lice in the Pacific Ocean show evidence of evolved resistance to parasiticide treatment
Source: Sci Rep. 2022 Mar 28;12:4775. doi: 10.1038/s41598-022-07464-1 (PMC8960799; doi:10.1038/s41598-022-07464-1)
Supplement: Supplementary file 1 — Supplementary Table S1. [file 41598_2022_7464_MOESM1_ESM.docx]

**Supplementary Information**

**Title:** Salmon lice in the Pacific Ocean show evidence of evolved resistance to parasiticide treatment

**Authors:** Sean C. Godwin, Andrew W. Bateman, Anna Kuparinen, Rick Johnson, John Powell, Kelly Speck, Jeffrey A. Hutchings

Table S1. The effective concentrations required to kill 50% of sea lice (EC_50_) for bioassays conducted in the Broughton Archipelago. “Farm ID” is the farm ID in Fig. 1 of the main text.

| **Assay ID** | **Date** | **Farm name** | **Farm ID** | **EC_50_: mean (ppb)** | **EC_50_: lower CI (ppb)** | **EC_50_: upper CI**  **(ppb)** | **Sex** | **Previous EMB treatment** |
| --- | --- | --- | --- | --- | --- | --- | --- | --- |
| 1 | 2010-09-28 | Wicklow Point | 5 | 167 | 124 | 222 | male | 0 |
| 1 | 2010-09-28 | Wicklow Point | 5 | 52 | 35 | 70 | female | 0 |
| 2 | 2011-11-06 | Midsummer Island | 10 | 135 | 109 | 172 | male | 0 |
| 2 | 2011-11-06 | Midsummer Island | 10 | 80 | 55 | 105 | female | 0 |
| 3 | 2012-02-14 | Wicklow Point | 5 | 342 | 303 | 390 | male | 1 |
| 3 | 2012-02-14 | Wicklow Point | 5 | 80 | 59 | 101 | female | 1 |
| 4 | 2012-12-18 | Wicklow Point | 5 | 216 | 193 | 246 | male | 0 |
| 4 | 2012-12-18 | Wicklow Point | 5 | 172 | 144 | 209 | female | 0 |
| 5 | 2013-10-16 | Wicklow Point | 5 | 195 | 171 | 225 | male | 1 |
| 5 | 2013-10-16 | Wicklow Point | 5 | 63 | 50 | 77 | female | 1 |
| 6 | 2013-12-16 | Potts Bay | 11 | 186 | 162 | 214 | male | 1 |
| 6 | 2013-12-16 | Potts Bay | 11 | 64 | 54 | 77 | female | 1 |
| 7 | 2014-01-13 | Midsummer Island | 10 | 193 | 161 | 236 | male | 0 |
| 7 | 2014-01-13 | Midsummer Island | 10 | 82 | 64 | 106 | female | 0 |
| 8 | 2014-01-15 | Glacier Falls | 7 | 236 | 192 | 294 | male | 0 |
| 8 | 2014-01-15 | Glacier Falls | 7 | 78 | 63 | 96 | female | 0 |
| 9 | 2014-01-27 | Doctor Islets | 15 | 171 | 139 | 214 | male | 1 |
| 9 | 2014-01-27 | Doctor Islets | 15 | 84 | 45 | 121 | female | 1 |
| 10 | 2014-04-28 | Port Elizabeth | 14 | 124 | 97 | 160 | male | 0 |
| 10 | 2014-04-28 | Port Elizabeth | 14 | 40 | 30 | 50 | female | 0 |
| 11 | 2014-10-06 | Wicklow Point | 5 | 205 | 168 | 257 | male | 0 |
| 11 | 2014-10-06 | Wicklow Point | 5 | 138 | 111 | 169 | female | 0 |
| 12 | 2014-11-03 | Midsummer Island | 10 | 146 | 109 | 186 | male | 1 |
| 12 | 2014-11-03 | Midsummer Island | 10 | 136 | 105 | 174 | female | 1 |
| 13 | 2015-05-05 | Port Elizabeth | 14 | 276 | 275 | 277 | male | 0 |
| 13 | 2015-05-05 | Port Elizabeth | 14 | 52 | 52 | 53 | female | 0 |
| 14 | 2016-01-04 | Potts Bay | 11 | 263 | 211 | 342 | male | 1 |
| 14 | 2016-01-04 | Potts Bay | 11 | 165 | 134 | 204 | female | 1 |
| 15 | 2016-05-09 | Doctor Islets | 15 | 132 | 106 | 165 | male | 0 |
| 15 | 2016-05-09 | Doctor Islets | 15 | 64 | 50 | 80 | female | 0 |
| 16 | 2016-11-15 | Wicklow Point | 5 | 254 | 206 | 322 | male | 0 |
| 16 | 2016-11-15 | Wicklow Point | 5 | 165 | 127 | 219 | female | 0 |
| 17 | 2017-01-10 | Burdwood | 6 | 321 | 253 | 419 | male | 0 |
| 17 | 2017-01-10 | Burdwood | 6 | 223 | 143 | 335 | female | 0 |
| 18 | 2017-09-25 | Wicklow Point | 5 | 260 | 206 | 332 | male | 1 |
| 18 | 2017-09-25 | Wicklow Point | 5 | 172 | 140 | 216 | female | 1 |
| 19 | 2018-02-14 | Midsummer Island | 10 | 271 | 225 | 336 | male | 0 |
| 19 | 2018-02-14 | Midsummer Island | 10 | 207 | 162 | 264 | female | 0 |
| 20 | 2018-06-04 | Port Elizabeth | 14 | 161 | 112 | 232 | male | 0 |
| 20 | 2018-06-04 | Port Elizabeth | 14 | 105 | 68 | 147 | female | 0 |
| 21 | 2018-06-14 | Port Elizabeth | 14 | 158 | 118 | 210 | male | 0 |
| 21 | 2018-06-14 | Port Elizabeth | 14 | 81 | 0 | 139 | female | 0 |
| 22 | 2019-03-27 | Burdwood | 6 | 437 | 284 | 667 | male | 0 |
| 22 | 2019-03-27 | Burdwood | 6 | 225 | 127 | 348 | female | 0 |
| 23 | 2019-04-02 | Sir Edmund Bay | 3 | 394 | 275 | 565 | male | 0 |
| 23 | 2019-04-02 | Sir Edmund Bay | 3 | 355 | 233 | 502 | female | 0 |
| 24 | 2019-05-07 | Wicklow Point | 5 | 66 | 47 | 85 | male | 0 |
| 24 | 2019-05-07 | Wicklow Point | 5 | 55 | 40 | 70 | female | 0 |
| 25 | 2019-05-08 | Cypress Harbour | 2 | 217 | 166 | 281 | male | 1 |
| 25 | 2019-05-08 | Cypress Harbour | 2 | 145 | 88 | 198 | female | 1 |
| 26 | 2020-01-08 | Midsummer Island | 10 | 307 | 219 | 432 | male | 0 |
| 26 | 2020-01-08 | Midsummer Island | 10 | 125 | 54 | 191 | female | 0 |
| 27 | 2020-08-31 | Wicklow Point | 5 | 338 | 213 | 472 | male | 0 |
| 27 | 2020-08-31 | Wicklow Point | 5 | 42 | 0 | 148 | female | 0 |
| 28 | 2021-02-02 | Doctor Islets | 15 | 498 | 395 | 641 | male | 0 |
| 28 | 2021-02-02 | Doctor Islets | 15 | 288 | 218 | 373 | female | 0 |
| 29 | 2021-04-26 | Port Elizabeth | 14 | 353 | 254 | 479 | male | 0 |
| 29 | 2021-04-26 | Port Elizabeth | 14 | 202 | 84 | 319 | female | 0 |
| 30 | 2021-07-13 | Wicklow Point | 5 | 907 | 744 | 1189 | male | 1 |
| 30 | 2021-07-13 | Wicklow Point | 5 | 840 | 695 | 1073 | female | 1 |
| 31 | 2021-07-20 | Swanson Island | 12 | 661 | 532 | 860 | male | 0 |
| 31 | 2021-07-20 | Swanson Island | 12 | 609 | 485 | 797 | female | 0 |
| 32 | 2021-10-05 | Swanson Island | 12 | 492 | 355 | 629 | male | 0 |
| 32 | 2021-10-05 | Swanson Island | 12 | 622 | 620 | 624 | female | 0 |
| 33 | 2021-04-20 | Larsen Island | 13 | 353 | 291 | 438 | male | 0 |
| 33 | 2021-04-20 | Larsen Island | 13 | 103 | 43 | 159 | female | 0 |
| 34 | 2021-08-09 | Midsummer Island | 10 | 557 | 469 | 678 | male | 0 |
| 34 | 2021-08-09 | Midsummer Island | 10 | 360 | 296 | 442 | female | 0 |
